# Antimony susceptible *Leishmania donovani*: evidence from *in vitro* drug susceptibility of parasites isolated from patients of post-kala-azar dermal leishmaniasis in pre- and post-miltefosine era

**DOI:** 10.1128/spectrum.04026-23

**Published:** 2024-05-07

**Authors:** Sushmita Ghosh, Aditya Verma, Dhiraj Kumar, Deepak Kumar Deep, V. Ramesh, Poonam Salotra, Ruchi Singh

**Affiliations:** 1 ICMR, National Institute of Pathology, Safdarjung Hospital Campus, New Delhi, India; 2 Department of Dermatology and STD, Safdarjung Hospital, Vardhman Mahavir Medical College, New Delhi, India; CSIR-Institute of Microbial Technology, Chandigarh, India

**Keywords:** multidrug resistance, *Leishmania*, post kala azar dermal leishmaniasis, antimony, miltefosine, amphotericin B

## Abstract

Post-kala-azar dermal leishmaniasis (PKDL) patients are a key source of *Leishmania donovani* parasites, hindering the goal of eliminating visceral leishmaniasis (VL). Monitoring treatment response and parasite susceptibility is essential due to increasing drug resistance. We assessed the drug susceptibility of PKDL isolates (*n* = 18) from pre-miltefosine (MIL) era (1997–2004) with isolates (*n* = 16) from the post-miltefosine era (2010–2019) and post-miltefosine treatment relapse isolates (*n* = 5) towards miltefosine and amphotericin B (AmB) at promastigote stage and towards sodium antimony gluconate (SAG) at amastigote stage. PKDL isolates were examined for mutation in gene-encoding AQP1 transporter, C26882T mutation on chromosome 24, and miltefosine-transporter (MT). PKDL isolates from the post-miltefosine era were significantly more susceptible to SAG than SAG-resistant isolates from the pre-miltefosine era (*P* = 0.0002). There was no significant difference in the susceptibility of parasites to miltefosine between pre- and post-miltefosine era isolates. The susceptibility of PKDL isolates towards AmB remained unchanged between the pre- and post-miltefosine era. However, the post-miltefosine era isolates had a higher IC_50_ value towards AmB compared with PKDL relapse isolates. We did not find any association between AQP1 gene sequence variation and susceptibility to SAG, or between miltefosine susceptibility and single nucleotide polymorphisms (SNPs in the MT gene. This study demonstrates that recent isolates of *Leishmania* have resumed susceptibility to antimonials *in vitro*. The study also offers significant insights into the intrinsic drug susceptibility of *Leishmania* parasites over the past two decades, covering the period before the introduction of miltefosine and after its extensive use.

**IMPORTANCE:**

Post-kala-azar dermal leishmaniasis (PKDL) patients, a key source of *Leishmania donovani* parasites, hinder eliminating visceral-leishmaniasis. Assessment of the susceptibility of PKDL isolates to antimony, miltefosine (MIL), and amphotericin-B indicated that recent isolates remain susceptible to antimony, enabling its use with other drugs for treating PKDL.

## INTRODUCTION

Drug-resistant *Leishmania* parasites constitute a significant threat to attain and sustain the elimination of visceral leishmaniasis (VL). Widespread resistance to antimony in *Leishmania* parasites was reported in early 2000, and the declining efficacy of miltefosine (MIL) has been witnessed with the decrease in drug susceptibility of parasites. Due to the high treatment failure rate, antimonials were replaced as the first-line drug for the treatment of visceral leishmaniasis (VL) and its sequel post-kala-azar dermal leishmaniasis (PKDL).

PKDL is a chronic disease that appears in 5-15% of treated VL patients in the Indian subcontinent (ISC). PKDL may also appear in individuals without any history of VL, and approximately 9-23% of patients reported in ISC do not have a history of it (1–4). PKDL patients represent a major reservoir of *Leishmania donovani* parasites, threatening the aim of reducing VL incidence to less than one case per 10,000 population.

Pentavalent antimonials (sodium antimony gluconate sodium antimony gluconate [SAG] or sodium stibogluconate) were once the primary treatment for VL and PKDL in ISC, but are now restricted due to treatment failures (5). PKDL treatment is closely linked to VL, and any substantial change in the treatment of VL would impact PKDL. Thus, MIL became the treatment of choice for VL and PKDL in ISC but was not recommended for pregnant or lactating women, children under 2 years old, or HIV-positive patients (6). Recently, in PKDL, there have been reports of relapses following MIL treatment (7). Amphotericin B (AmB) is recommended as a second-line treatment option for patients who are unresponsive to the first-line treatment, MIL, or who experience adverse side effects or teratogenicity, as per the NVBDCP guidelines (6). Ramesh et al. (8) have suggested a combination regimen of MIL and liposomal AmB as an effective treatment for PKDL (8).

As a good regimen has yet to be evolved, monitoring the susceptibility of PKDL isolates needs to be stepped up. A study by Singh et al. (9) found a strong correlation between clinical response and *in vitro* susceptibility of VL and PKDL isolates to SAG (9). In addition to the monitoring of treatment outcomes and *in vitro* susceptibility of PKDL clinical isolates, it is crucial to establish early detection techniques to differentiate drug-resistant parasites. While some genes linked to drug resistance in *Leishmania* have been recognized, whether they can serve as biomarkers for monitoring drug resistance requires further studies. Previous research indicates that antimonial resistance may be linked to a truncated protein or down-regulation of aquaporin 1 (AQP1), which is responsible for antimonial drug uptake (10, 11). Additionally, a nonsynonymous mutation located at position 26882 on chromosome 24, which encodes ankyrin/TPR repeat protein, has been suggested to be associated with antimonial resistance (12, 13). Similarly, resistance to MIL has been connected to mutations in the genes that encode the MIL transporter (MT) and its β-subunit (Ros3) (14, 15).

In this study, we assessed the susceptibility of PKDL isolates (*n* = 34) obtained during pre- and post-MIL era towards SAG, MIL, and AmB. Additionally, we evaluated the *in vitro* susceptibility of a few isolates (*n* = 5) from PKDL cases that relapsed after MIL treatment, obtained between 2010 and 2016. Furthermore, we aimed to analyse selected genes that can be used as molecular tools for monitoring drug resistance. For this purpose, we evaluated single nucleotide polymorphism (SNP) in the gene encoding AQP1 and the ankyrin/TPR repeat protein, which may be responsible for altered SAG susceptibility in various isolates. We also carried out sequence analysis of the gene encoding MT to identify SNPs in PKDL isolates.

## MATERIALS AND METHODS

### Patients and parasite culture

*L. donovani* isolates were prepared from dermal lesions of PKDL patients who reported to the Department of Dermatology, Safdarjung Hospital, New Delhi in pre-MIL (1997–2004) and post-MIL (2011–2019) periods. PKDL relapse cases approached the hospital with the appearance of macular or papular lesion after they were cured of PKDL lesion after treatment with miltefosine. All the patients who reported with lesions were confirmed for the presence of parasite by histopathology or QPCR assay as described earlier (8). The skin biopsy samples (3 mm punch biopsy) of dermal lesions from PKDL and PKDL relapse patients were collected under aseptic conditions and placed in M199 medium (Sigma-Aldrich, St. Louis, MO, USA) with HEPES (pH 7.4), supplemented with 10% heat-inactivated FBS (HI FBS, Gibco, Waltham, MA, USA), 100 IU penicillin G, 100 µg/mL of streptomycin, and a mixture of vitamins and amino acids, and incubated at 26°C in a BOD incubator. Cultures with microscopically visible *Leishmania* promastigote was further propagated and maintained in M199 medium for 2–3 weeks; and once parasite culture become stable, it was cryopreserved in liquid nitrogen as described elsewhere (16). Briefly, late log phase parasites in their initial passages were washed and cryopreserved in cryovials at a final concentration of 1 × 10^8^ promastigotes/mL in M199 medium supplemented with 20% heat-inactivated FBS and 5% (v/v) DMSO. The cryovials were placed in a cryochil cooler with isopropanol, transferred to −70°C for 24 hours, and then stored in the vapor phase of liquid nitrogen for future use.

### *In vitro* drug susceptibility assay

Determining drug susceptibility against the intracellular amastigote stage of *L. donovani* is the most reliable method for antimonials, as promastigotes show lower susceptibility, which does not correlate with the amastigote stage (9, 17, 18). However, in the case of both AmB and MIL, the susceptibility of promastigotes and amastigotes is comparable and exhibits a strong correlation in *L. donovani* (17, 19). Based on these findings, a simpler and more accessible promastigote assay was used for *in vitro* susceptibility testing of MIL and AmB drugs, as there is a strong inter-stage correlation. For SAG, the intracellular amastigote assay was used to determine the *in vitro* drug susceptibility.

#### Promastigote assay

We evaluated the susceptibility of PKDL field isolates towards MIL and AmB using the resazurin-based fluorometric assay as previously described (19). In 96 well plates, log phase promastigotes (5 × 10^5^ parasites/well) were incubated in 200 µL complete medium or serial dilutions of (i) MIL at concentrations ranging from 0.156 to 160 µM or (ii) AmB at concentrations ranging from 0.009 to 10 µg/mL. The concentration range was selected based on the previously reported susceptibility range for the *Leishmania* parasites (17–20). Cell viability was measured fluorimetrically, and the percentage reduction in parasite viability was determined by comparing treated wells with untreated controls. Fifty percent inhibitory concentration (IC_50_) was calculated using sigmoidal regression analysis.

#### Amastigote assay

SAG susceptibility at the intracellular amastigote stage was evaluated *in vitro* using mouse macrophage adherent cell line J774A.1. The concentration range tested for SAG was from 1 to 60 µg/mL. This range was selected based on the reported drug susceptibility range for L. donovani parasites in previous studies. Concentrations higher than 100 µg/mL of SAG were found to be cytotoxic to host cells (9, 20). Briefly, macrophage cells, J774A.1, were seeded at 2 × 105 cells/well in 8-well chamber slides (Nunc) and left to adhere. The medium was gently removed after 24 h, and macrophages were infected with 10 promastigotes per macrophage in 400 µL complete RPMI 1640 medium. After 24 h, infected macrophages were treated with SAG (1, 5, 10, 20, 40, and 60 µg/mL) for 48 h. Intracellular amastigotes were counted in 100 macrophages at a magnification of 1000× after staining with Diff-Quik solutions. IC_50_ value was determined based on the parasite survival rate compared with untreated macrophages.

### SNP detection at AQP1 gene, the region containing position 26882 on chromosome 24, and MT gene

Mutations in gene encoding AQP1 transporter (*LinJ.31.0030*) and C26882→T mutation on chromosome 24 at position 26882 implicated in SAG resistance and miltefosine transporter LdMT were examined in PKDL isolates (pre-treatment and relapse). Primers and PCR reaction conditions are listed in Table S1 in the supplementary material. The genes were amplified using gene-specific primers, and sequence was determined. Sequences obtained for 18 PKDL clinical isolates were aligned using Clustal 2.1 with reference strain *L. donovani* BPK282/0cl4 used for AQP1 and Ankyrin/TPR repeat gene while MHOM/IN/1983/AG83 was used as a reference for LdMT gene sequence analysis.

### Statistical analysis

The statistical analysis was conducted using GraphPad Prism 5 software (GraphPad Software Inc., San Diego, CA, USA). The Mann-Whitney test was used to determine statistical significance, with *P* values < 0.05 considered significant.

## RESULTS

Drug susceptibility of PKDL-derived *L. donovani* isolates from pre-miltefosine era (*n* = 18), post-miltefosine era (*n* = 16), and relapse (*n* = 5) isolates was determined towards miltefosine and amphotericin B at promastigote stage and towards SAG at amastigote stage. The patients from pre-MIL era were treated with SAG, whereas those from post-MIL era were treated with either MIL monotherapy or liposomal AmB (LAmB) and MIL combination therapy. The isolate name, year of isolation, treatment, and their *in vitro* IC_50_ ±SD values are summarized in Tables 1 to 3.

**TABLE 1:** Susceptibility of PKDL patient derived *Leishmania* isolates (1996–2004) towards various anti-leishmanial drugs^*a*^

S.no.	Isolate name and year of isolation	Year of VL (history of VL in y)	Treatment^*b*^ PKDL	Response to treatment	MIL IC_50_ ±SD (µM) promastigote	AmB IC_50_ ±SD (µg/mL) promastigote	SAG IC_50_ ±SD (µg/mL) amastigote
1	P21/1997	1996 (1)	SAG	Responsive	3.18 ± 0.62	0.96 ± 0.08	6.4 ± 0.33
2	P44/1998	1995 (3)	SAG	Responsive	1.27 ± 0.16	0.57 ± 0.04	6.3 ± 0.24
3	P48/1998	No history	SAG	ND	1.21 ± 0.57	0.71 ± 0.12	31.2 ± 4.5
4	P49/1999	1996 (3)	SAG	Slow Response	1.65 ± 0.64	0.88 ± 0.21	20 ± 2.64
5	P56/1999	1995 (4)	SAG	Responsive	3.23 ± 0.21	0.77 ± 0.11	5.5 ± 1.19
6	P69/1999	1994 (5)	SAG	Slow Response	3.3 ± 0.24	1.68 ± 0.23	17.9 ± 3.32
7	P75/2000	1996 (4)	SAG	Responsive	2.42 ± 0.11	1.18 ± 0.25	3.9 ± 0.42
8	P82/2000	1999(0.5)	SAG	Responsive	3.4 ± 0.26	0.98 ± 0.34	4.3 ± 0.32
9	P83/2000	1993 (7)	SAG	Relapsed	2.53 ± 0.19	0.92 ± 0.27	28.7 ± 1.02
10	P84/2000	1992 (8)	SAG	Responsive	3.77 ± 0.34	1.11 ± 0.12	4.2 ± 0.56
11	P85/2000	1994 (6)	SAG	Slow Response	2.52 ± 0.21	0.53 ± 0.06	8.5 ± 0.78
12	P86/2000	1985 (15)	SAG	ND	3.04 ± 0.11	0.57 ± 0.08	4.8 ± 0.23
13	P93/2001	1993 (8)	SAG	Responsive	1.76 ± 0.31	0.76 ± 0.16	2.9 ± 0.13
14	P94/2001	1998 (3)	SAG	ND	2.13 ± 0.45	0.9 ± 0.11	15.3 ± 2.98
15	P100/2002	1999 (3)	SAG	Responsive	2.54 ± 0.19	0.86 ± 0.15	3 ± 0.41
16	P101/2002	1993 (9)	SAG	ND	5.12 ± 0.64	1.94 ± 0.24	36 ± 3.35
17	P136/2003	2000 (3)	SAG	Slow Response	4.62 ± 0.14	0.93 ± 0.11	12.4 ± 2.03
18	P137/2004	2001 (3)	SAG	Responsive	3.33 ± 0.29	1.04 ± 0.28	4.7 ± 1.04

^*a*^ SAG - sodium antimony gluconate; NA - not available; ND - not determined; PKDL - post-kala-azar dermal leishmaniasis.

^*b*^ All these PKDL patients were treated with SAG during the VL episode.

**TABLE 2:** Susceptibility of PKDL patient derived *Leishmania* isolates (2011–2019) towards various anti-leishmanial drugs^*a*^

S.no.	Isolate name and year of isolation	Year of VL (history of VL in y)	Interval between VL and PKDL appearance (y)	Treatment VL	Treatment PKDL	Response to treatment	MIL IC_50_ ±SD (µM) promastigote	AmB IC_50_ ±SD (µg/mL) promastigote	SAG IC_50_ ±SD (µg/mL) amastigote
1	P232/2011	2005 (6)	4	Amphotericin B	MIL	Responsive	4.28 ± 0.38	1.34 ± 0.007	8.75 ± 1.23
2	P235/2011	1996 (15)	2	SAG	MIL	Relapsed and cured with AmB	6.37 ± 1.44	1.36 ± 0.006	9.62 ± 0.73
3	P240/2012	2003 (9)	3	SAG for 1mo	MIL	ND	3.44 ± 0.03	1.66 ± 0.003	7.84 ± 1.47
4	P241/2012	No history	NA		MIL	Responsive	7.55 ± 0.67	1.65 ± 0.07	8.65 ± 0.43
5	P260/2013	2011 (2)	2	SAG	MIL	Responsive	0.920.004	0.185 ± 0.007	9.43 ± 1.22
6	P261/2013	No history	NA	-	MIL	Responsive	1.27 ± 0.35	0.225 ± 0.007	9.42 ± 0.89
7	P262/2013	No history	NA	-	MIL	Relapsed; cured with Amphomul	1.58 ± 0.32	0.23 ± 0.03	11.23 ± 2.15
8	PK41/2015	2000 (15)	10	NA	MIL	Relapsed; cured with LAmB	1.92 ± 0.07	1.22 ± 0.004	6.57 ± 0.32
9	PK52/2015	2000 (15)	1	NA	MIL +LAmB	Responsive	1.83 ± 0.3	1.37 ± 0.003	3.8 ± 0.08
10	PK85/2018	NA	NA	NA	MIL +LAmB	Responsive	2.14 ± 0.01	1.69 ± 0.05	4.2 ± 0.21
11	PK86/2018	1993 (25)	0.25	SAG	MIL +LAmB	Intolerant to LAmB; Lost to follow up	1.29 ± 0.12	0.55 ± 0.017	4.62 ± 0.49
12	PK96/2018	2016 (2)	2	NA	MIL +LAmB	Responsive	7.68 ± 0.9	2.2 ± 0.002	2.9 ± 0.42
13	PK100/2019	1999 (20)	10	NA	MIL +LAmB	Responsive	4.06 ± 0.62	2 ± 0.148	3.11 ± 0.05
14	PK103/2019	2008 (11)	10	NA	MIL +LAmB	Responsive	2.82 ± 0.3	1.13 ± 0.0002	4.51 ± 0.098
15	PK106/2019	1999 (20)	12	SAG	MIL +LAmB	ND	2.7 ± 0.42	2.2 ± 0.007	4.74 ± 0.05
16	PK107/2019	No history	NA	NA	MIL +LAmB	Responsive	3.9 ± 0.27	4 ± 0.007	5.39 ± 0.03

^*a*^ MIL - miltefosine; LAmB - liposomal amphotericin B; SAG - sodium antimony gluconate; NA - not available; ND- not determined; PKDL - post-kala-azar dermal leishmaniasis.

**TABLE 3:** Susceptibility of PKDL relapse isolates towards various anti-leishmanial drugs

S.no.	Isolate name	Year of isolation	Treatment	MIL IC_50_ ±SD (µM) promastigote	AmB IC_50_ ±SD (µg/mL) promastigote	SAG IC_50_ ±SD (µg/mL) amastigote
1	P214 R	2010	MIL	16.85 ± 0.636	2.2 ± 0.35	18.4 ± 2.12
2	P228R	2012	MIL	9.36 ± 1.21	2.1 ± 0.01	19.98 ± 1.95
3	P270R	2015	MIL	16.19 ± 1.6	2.5 ± 0.02	3.2 ± 0.2
4	PK34R	2016	LAmB	16.89 ± 1.08	0.8 ± 0.018	7.54 ± 0.4
5	PK274R	2016	LAmB	17.61 ± 0.125	1.1 ± 0.028	6.85 ± 0.5

### SAG susceptibility

**Fig 1:**
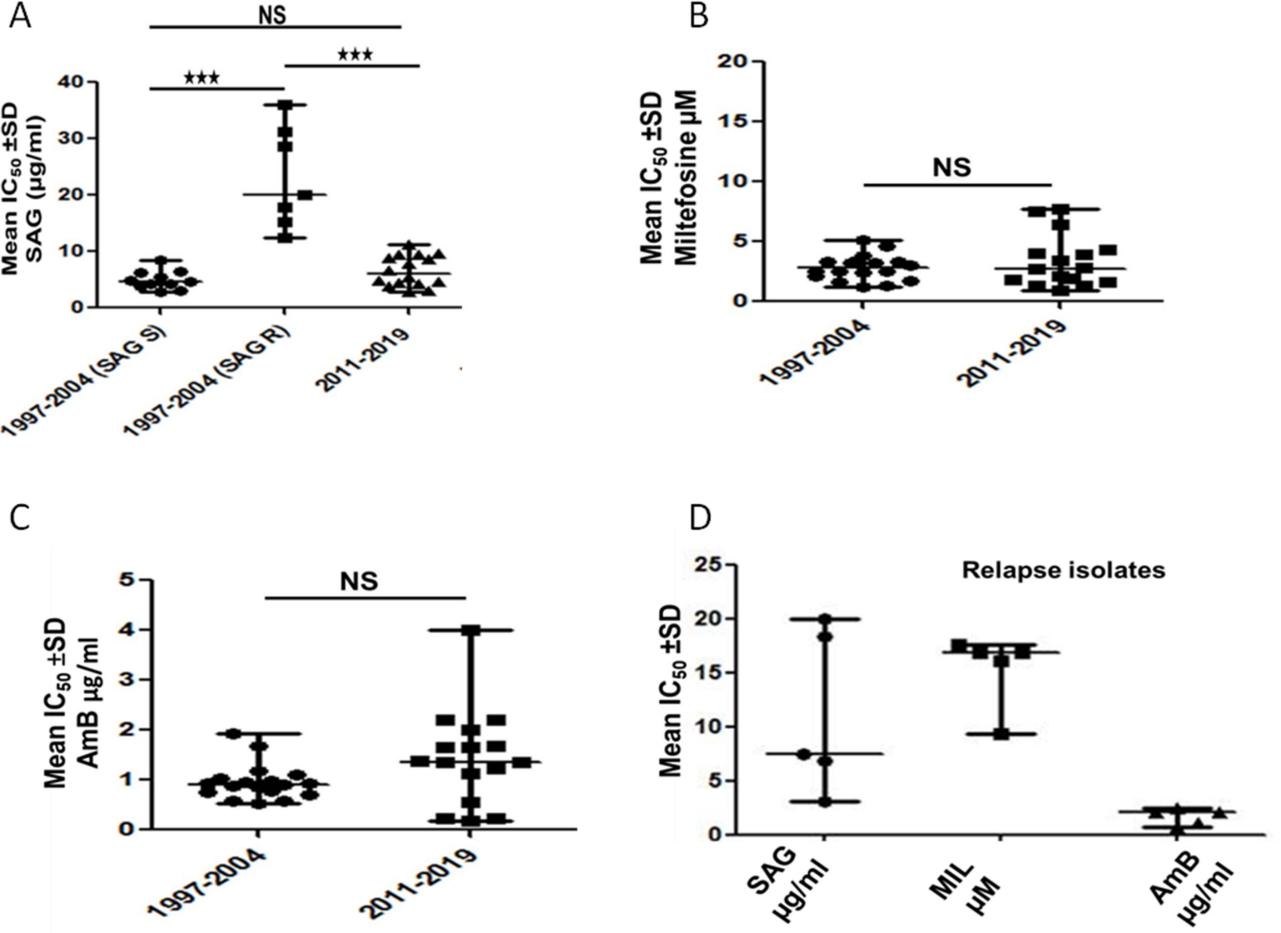
*In vitro* of drug susceptibility of *Leishmania* parasites isolated from pre-treatment PKDL and PKDL-relapse patients for various drugs. Susceptibility of pre-treatment isolates to (**A**) SAG (at intracellular amastigote stage). The isolates from post MIL era showed a significant increase in drug susceptibility (*P* = 0.0002), (**B**) MIL (promastigote stage). Susceptibility towards MIL did not change significantly in pre- and post- MIL era (**C**) Am B (promastigote stage). Susceptibility towards Am B did not change significantly in pre- and post- MIL era (**D**) SAG, MIL, and AmB susceptibility in PKDL-relapse isolates. MIL - miltefosine; SAG - sodium antimony gluconate; NS = non-significant; *P* > 0.05; ***= *P* ≤ .001.

The isolates from pre-MIL era (*n* = 18) showed an IC_50_ range of 2.9 ± 0.13 to 36 ± 3.35 µg/mL towards SAG. These isolates were categorized as SAG sensitive (SAG-S) and SAG resistant (SAG-R) isolates according to the criteria given by Singh et al.(9). SAG-S isolates (*n* = 11) showed IC_50_ values from 2.9 ± 0.13 µg/mL to 8.5 ± 0.78 µg/mL with mean and median IC_50_ values of 4.95 ± 1.6 µg/mL and 4.7 ± 1.04 µg/mL, respectively. SAG R (*N* = 7) isolates showed IC_50_ range from 12.4 ± 2.03 µg/mL to 36 ± 3.35 µg/mL with mean and median values of 23.07 ± 8.55 µg/mL and 20 ± 2.64 µg/mL, respectively (Table 1). Thus, there was a significant increase of 4.26 fold (*P* = 0.0006) in the median IC_50_ of SAG-R isolates compared with SAG-S isolates (Fig. 1A).

In the post-MIL era, the isolates (*n* = 16) showed IC_50_ values ranging from 2.9 ± 0.42 µg/mL to 11.23 ± 2.15 µg/mL with a mean and median value of 6.55 ± 2.66 µg/mL and 5.98 ± 3.51 µg/mL, respectively (Table 2).

Comparison of IC_50_ between pre- and post-MIL era revealed a significant decrease (*P* = 0.0002) of 3.34 fold in the median IC_50_ of isolates from post-MIL era than SAG-R isolates from pre-MIL era. However, there was no significant difference in the mean IC_50_ of SAG-S isolates from pre-MIL era and that of isolated from post-MIL era (Fig. 1A).

### MIL susceptibility

All 18 isolates from the pre-MIL era exhibited IC_50_ values towards MIL ranging from 1.21 ± 0.57 µM to 5.12 ± 0.64 µM, with a mean IC_50_ of 2.83 ± 1.04 µM and a median IC_50_ of 2.79 ± 0.88 µM (Table 1). Isolates of post-MIL era exhibited a range of IC_50_ values from 0.92 ± 0.004 µM to 7.68 ± 0.9 µM, with a mean of 3.35 ± 2.14 µM and median of 2.76 ± 1.69 µM (Table 2). The IC_50_ values of isolates from both pre-MIL and post-MIL era were not significantly different towards MIL (Fig. 1B).

### AmB susceptibility

The IC_50_ for AmB of 18 isolates from the pre-MIL era and 16 isolates from the post-MIL era ranged from 0.53 ± 0.06 µg/mL to 1.94 ± 0.24 µg/mL and 0.185 ± 0.007 to 4.0 ± 0.007 µg/mL, respectively (Table 1). The mean and median IC50 values in the pre-MIL era isolates were 0.96 ± 0.35 µg/mL and 0.91 ± 0.20 µg/mL, respectively. In the post-MIL era isolates, the mean and median IC_50_ values increased to 1.44 ± 0.94 µg/mL and 1.365 ± 0.74 µg/ml, respectively (Table 2). The mean IC_50_ of isolates from the post-MIL era did not significantly differ from those of the pre-MIL era (Fig. 1C).

### Susceptibility of relapse isolates

The susceptibility of a small number of PKDL relapse isolates (*n* = 5) collected from patients between 2010 and 2016 was tested against SAG, MIL, and AmB. For SAG, the IC_50_ values ranged from 3.2 ± 0.2 µg/mL to 19.98 ± 1.95 µg/mL, with mean and median values of 11.19 µg/mL and 7.54 µg/mL, respectively. It was observed that the relapse isolates collected during 2010–2012 showed higher IC_50_ values for SAG than the isolates from 2015 to 2016 (Table 3). The IC_50_ values for MIL ranged from 9.36 ± 1.21 µM to 17.61 ± 0.125 µM, with mean and median values of 15.38 ± 3.20 µM and 16.85 ± 0.63 µM. For AmB, the IC_50_ values ranged from 0.8 ± 0.018 µg/mL to 2.5 ± 0.02 µg/mL. The mean and median IC_50_ values for AmB were 1.74 ± 0.70 µg/mL and 2.1 ± 0.01 µg/mL, respectively (Fig. 1D).

### Comparative drug susceptibility of pre-treatment vs relapse isolates from pre- and post-miltefosine era

The susceptibility of pre- and post-MIL era isolates to SAG, MIL, and AmB was compared. For SAG, no significant difference was observed in the median IC_50_ of PKDL relapse isolates when compared with that of SAG-S or SAG-R isolates from pre-MIL era. Interestingly, no significant variation was observed in the median IC_50_ of PKDL relapse isolates compared with pre-treatment isolates obtained in the post-MIL era.

We observed a significant increase in the median IC_50_ of PKDL relapse isolates for MIL by 6.04 fold (*P* = 0.0009) compared with pre-MIL era isolates, and a 6.1 fold increase (*P* = 0.001) compared with post-MIL era isolates. For AmB, a significant increase (2.30 fold, *P* = 0.033) in the mean IC_50_ of PKDL relapse isolates was observed as compared with pre-treatment isolates in the pre-MIL era.

### Sequence analysis of AQP1 gene, the region containing position 26882 on chromosome 24 and MT gene

AQP1 gene sequence analysis in PKDL isolates (*n* = 18), including 13 pre-treatment and five relapse isolates, revealed genetic variation in AQP1 gene in the form of TC nucleotide insertion at 469–70 position within three isolates (two pre-treatment and one relapse) with respect to the reference isolate. This resulted in the formation of 218 amino acid truncated protein as compared with references *L. donovani* AQP1 having 314 amino acids (Fig. S1A). AQP1 gene sequence was unaltered in the remaining 15 isolates. Genetic variation observed in AQP1 did not appear to be associated with SAG susceptibility in these isolates.

Two pre-treatment isolates (P241 and PK103) and one relapse isolate (P214R) showed C26882→T mutation. Although the mutation was identified in two pre-treatment isolates, neither of them showed a higher IC_50_ for SAG. However, the isolate obtained after relapse (P214R) following miltefosine treatment showed a higher IC_50_ for SAG (Fig. S1B).

No T527A and C1259A mutations could be detected in the putative *L. donovani* miltefosine transporter for any pre-treatment and relapse isolate (Fig. S1C and D), indicating that the variable susceptibility of these isolates to MIL was not influenced by the MT gene sequence.

## DISCUSSION

PKDL is a potential reservoir for the spread of VL in the ISC region (21). In order to sustain the elimination of VL in the ISC, it is essential to consider the inherent variation in the susceptibility of *L. donovani* parasites isolated from different patients. The current study comprehensively analyzed 34 PKDL isolates and their susceptibility to SAG, MIL, and AmB in a controlled laboratory environment. *Leishmania* parasites isolated from PKDL patients were collected over a 23-year period, allowing us to compare the susceptibility patterns before and after the introduction of MIL. The study also examined parasites obtained from patients who relapsed after MIL treatment.

Most forms of leishmaniases have been successfully treated with pentavalent antimonials; however, in recent years, treatment failures in endemic areas have discouraged the use of these drugs in ISC (5). In this study, we observed that PKDL isolates from the post-MIL era were significantly more susceptible to SAG than SAG-R isolates from the pre-MIL era; the isolates were derived from PKDL cases who had VL much before the introduction of MIL. Upon comparing PKDL relapse isolates to pre-treatment isolates from pre-MIL and post-MIL eras, our analysis revealed no significant alteration in susceptibility towards SAG and MIL. Of the 16 PKDL isolates collected in the post-MIL era, four did not have a history of VL, and only three had VL episodes after the introduction of MIL for treatment in India. Three of the four isolates with no history of VL were from the years 2012 and 2013 and exhibited high IC_50_ for SAG but less than the cut-off of 11.0 µg/mL (9, 22). One isolate with no VL history was isolated in 2019 and exhibited comparatively low IC_50_ of 5.39 ± 0.03 µg/mL. Of the three isolates collected post 2004, one was treated with SAG during VL and displayed high IC_50_ of 9.43 ± 1.22 µg/mL, and two other isolates exhibited IC_50_ of 4.74 ± 0.05 and 2.9 ± 0.42 µg/mL, these results indicate that SAG susceptible parasites are in circulation in the endemic region and exposure to the drug during VL treatment contributes to increased drug tolerance.

MIL is the only effective oral treatment for PKDL with limited toxicity, which includes major gastrointestinal side effects, nephrotoxicity, hepatotoxicity, and teratogenicity. Despite its effectiveness, there have been reports of relapses following MIL treatment, and its efficacy has dipped to 85% (7). A recent study from India on PKDL patients treated with MIL in India has raised serious concerns about the safety and efficacy of the treatment. The study reported final cure rates of 76%, but also highlighted ocular complications in a significant number of cases (23). In the present study, no significant difference was observed in the susceptibility of *L. donovani* parasites to MIL between pre- and post-MIL era isolates, indicating no change in their natural susceptibility towards this drug. However, the significant decrease in susceptibility of PKDL relapse isolates compared with pre- and post-MIL era isolates indicates that the emergence of drug-resistant parasites cannot be avoided.

AmB liposomal is a safe and effective alternative treatment for ISC but requires proper monitoring to prevent drug resistance (24, 25). This study found that the susceptibility of PKDL isolates towards AmB remained unchanged between the pre-and post-MIL era. However, post-MIL era isolates had a higher IC_50_ value towards AmB that was comparable to PKDL relapse isolates. In contrast, pre-MIL era isolates had significantly higher susceptibility than PKDL relapse isolates. It is alarming to see a shift in susceptibility toward AmB, signaling the emergence of AmB- tolerant *Leishmania* parasites.

The AQP1 protein in *L. donovani* plays a crucial role in the transport of water and solutes. The reduced expression of AQP1 has been found to correlate with SAG resistance in parasites, and gene sequence variation has also been associated with SAG susceptibility (11). Our study found no association between AQP1 gene sequence variation and susceptibility to SAG, indicating it is not a suitable marker for further exploration. We also did not find any correlation between SAG susceptibility and a previously proposed C-T mutation within the gene encoding ankyrin/TPR repeat protein for SAG resistance (13). Similarly, we did not observe any mutations in the gene encoding MT in PKDL isolates, either pre-treatment or relapse, and no association was found between susceptibility towards MIL and SNPs in MT (14). These findings highlight the need for further investigation to identify new markers for drug resistance.

The results indicate the presence of SAG-susceptible *L. donovani* parasites in the endemic region. Additionally, PKDL cases, which are known to serve as a source of parasites during the transmission of VL disease, may also be transmitting SAG-susceptible parasites. Conducting further research on *Leishmania* isolates obtained from VL cases can provide more evidence of the circulation of SAG-susceptible *Leishmania* in endemic regions. Timely identification and treatment of PKDL are crucial to eliminate these reservoirs and prevent future epidemics of VL from re-emerging. It is imperative to continuously monitor the response to treatment and behavior of *Leishmania* parasites in order to achieve a sustained elimination of VL from ISC.

This study offers significant insights into alterations in the innate behavior of *L. donovani* field isolates spanning two decades. The insights gathered will play a vital role in adopting treatment strategies to address and contain the spread of leishmaniasis effectively. It’s important to note that SAG resistance developed due prolonged and high-volume drug injection, preventing patients from completing recommended treatment. However, by using SAG in combination with miltefosine or other drugs, we can potentially improve the efficacy of the treatment for PKDL and help eradicate the infection’s reservoir. The approach of using SAG in combination therapy with other drugs could be a valuable addition to the treatment of PKDL.

## Data Availability

The nucleotide sequences of various genes of *L. donovani* isolates studied in this study were submitted at GenBank nucleotide sequence database under the following accession numbers: Ankyrin/TRP repeat gene (PP598036–PP598053), miltefosine transporter LdMT_(T527A)_ (PP598073– PP598089) and LdMT_(C1259A)_ (PP598054–PP598072) and AQP1 gene (PP598090-PP598107).

## SUPPLEMENTAL MATERIAL

Supplementary material spectrum.04026-23-s0001.docx Fig. S1; Table S1.

ASM does not own the copyrights to Supplemental Material that may be linked to, or accessed through, an article. The authors have granted ASM a non-exclusive, world-wide license to publish the Supplemental Material files. Please contact the corresponding author directly for reuse.
