# Supplementary material for: Antimony susceptible Leishmania donovani: evidence from in vitro drug susceptibility of parasites isolated from patients of post-kala-azar dermal leishmaniasis in pre- and post-miltefosine era
Source: Microbiol Spectr. 2024 May 7;12(6):e04026-23. doi: 10.1128/spectrum.04026-23 (PMC11237457; doi:10.1128/spectrum.04026-23)
Supplement: Supplementary material — Fig. S1; Table S1. [file spectrum.04026-23-s0001.docx]

**PCR amplification and sequencing of AQP1, Ankyrin/TPR repeat, and MIL transporter gene for SNP detection.**

The association of SNPs present in AQP1, Ankyrin/TPR repeat and LdMT, gene was investigated in 18 PKDL isolates (13 pre-treatment and 5 relapse isolates) using PCR amplicons DNA sequence analysis. The AQP1, Ankyrin/TPR repeat and LdMT gene-specific primers were designed, and PCR reaction was performed on Applied Biosystems Proflex PCR System. The primer utilized for PCR amplification of selected genes is represented in **Table S1.**

**Table S1: Primer used to examine the single-nucleotide polymorphisms in LdMT, AQP1 and Ankyrin/ TPR repeat genes**

| **Nucleotide position** | **Chr. no.** | **LdMT Primer sequence (5'🡪3')** | **Amplicon size** | **Gene Accession No.** | |
| --- | --- | --- | --- | --- | --- |
| _527_T →A | **13** | **F 5'-**ATGCCCAACCAACCGCCGTGTTGG- **3'**  **R 5'-**TACAGGAACTGGTTCAGCGAGAGAG-**3'** | **769** | **AY321297.1** | |
| _1259_C →A | **13** | **F 5'**-TAGCCATCCTCATATTCCAGAAC -**3'**  **R 5'**-TGTGCCCCTGCACATCCGGGGCGC -**3'** | **840** | **AY321297.1** | |
| **AQP1 Primer sequence (5'🡪3')** | | | | | |
| TC insertion | **31** | **F 5'**-ATGAACTCTCCTACAAGCACAC-**3'**  **R 5'**-CTAGAAGTTGGGTGGAA TGATG-**3'** | **945** | | **FR796463.1** |
| **Ankyrin/TPR gene present on chromosome 24 position 26884; Primer sequence (5'🡪3')** | | | | | |
| _26882_C→T | **24** | **F 5'**-GACAGCTGGAGTGCATTCGC-**3'**  **R 5'**-CTTGCCCTCTCCGTTCTTGG-**3'** | **473** | | **FR799611.1** |

PCR reaction mixture was prepared for 50µl reaction volume, containing 1X *Taq* buffer, 4 mM MgCl_2_, 0.2 mM deoxynucleotide triphosphate (dNTP) mix, 50 ng of each sense and antisense primers, 3U of *Taq* DNA polymerase (Bangalore Genie, India) genomic DNA (100 ng), and final volume make up with nuclease-free water. The thermal cycling reaction conditions used to perform PCR were:

1. AQP1: Initial denaturation at 94°C for 5 min succeeded by 30 cycles of denaturation at 94°C for 45 sec, annealing at 65°C for 30 sec., extension at 72°C for 45 sec., and final extension for 5 min at 72°C.
2. Ankyrin/TPR repeat gene: initial denaturation for 5 min at 95 °C followed by denaturation for 30 sec at 94 °C, annealing at 59.8 °C for 30 sec, extension for 45 sec at 72 °C for 30 cycles and final extension at 72 °C for 10 minutes.
3. LdMT: Initial denaturation for 2 minutes at 94°C succeeded by 30 cycles of denaturation at 94°C for 45 sec., annealing at 45sec., extension at 68°C for 1min and final extension at 68°C for 3 min.

**A. TC insertion AQP1**

**B. _26882_C→T Ankyrin/TRP**


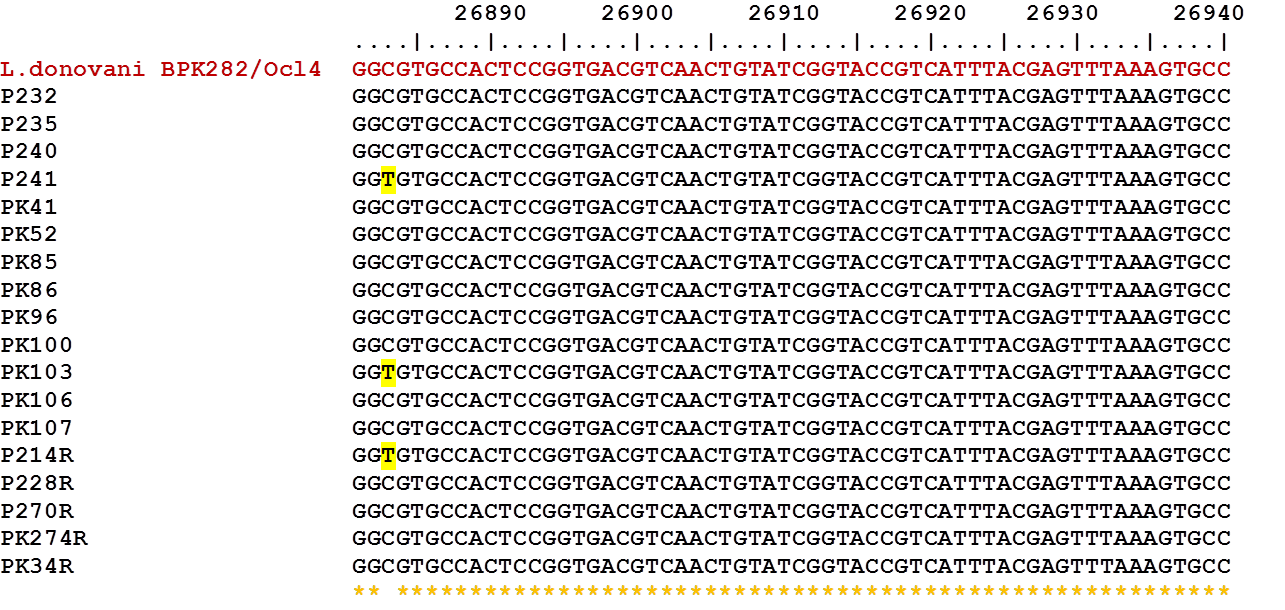


**C. Mutation _527_T→A LdMT**


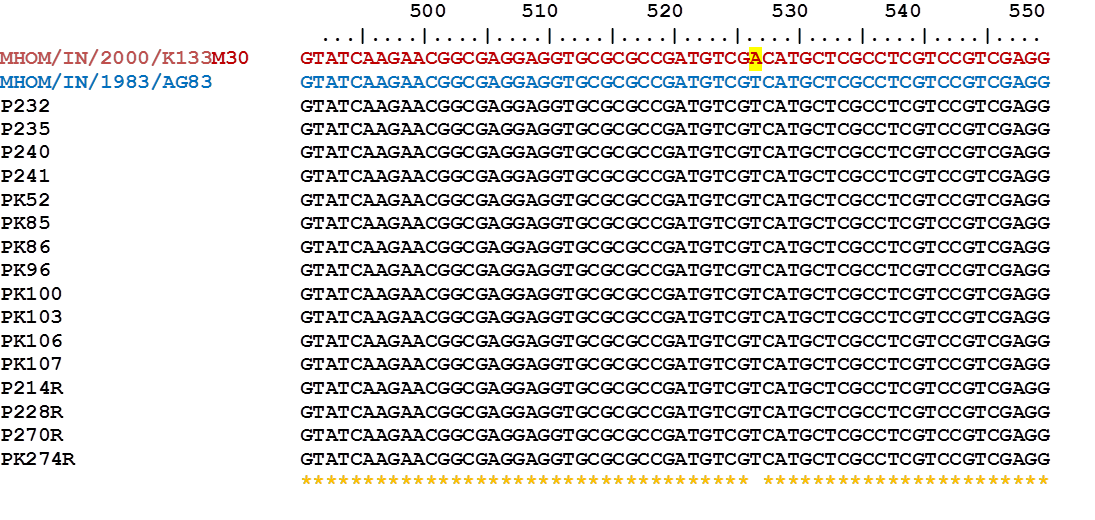


**D. Mutation _1259_A→C LdMT**


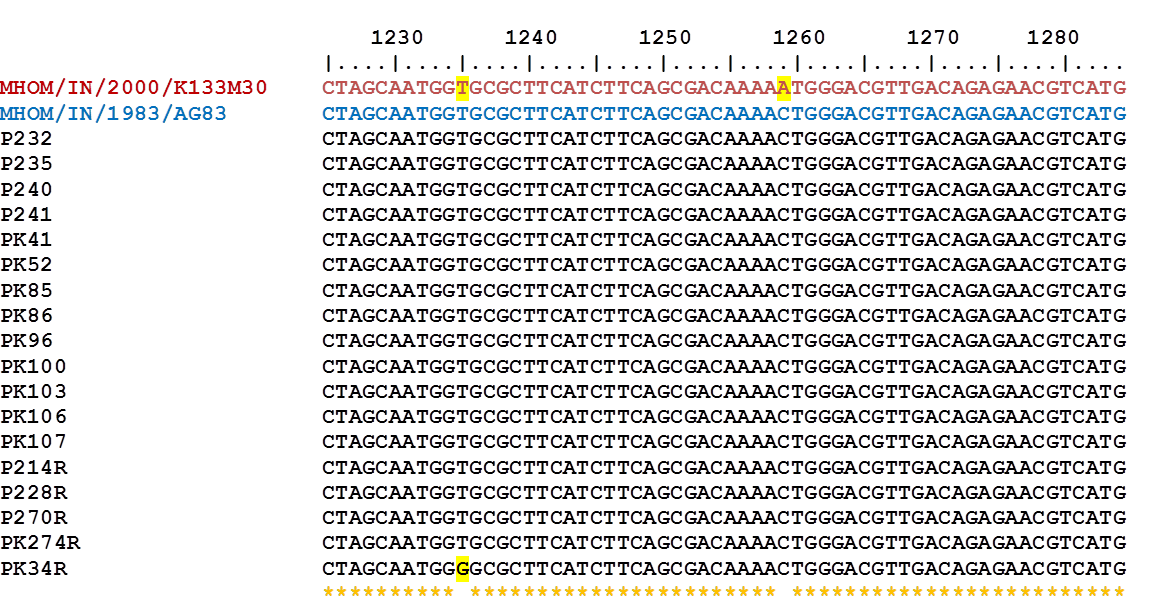


**Figure S1. Multiple sequence alignment by CLUSTAL omega (1.2.4) showing the genetic variation of sequence in different PKDL and PKDL relapse clinical isolates** **at gene level compared to *L. donovani* reference (A) AQP1 (B) region 26882 at chromosome 24 (C) LdMT1 (D) LdMT3**. (A) Sequence alignment for TC insertion in the AQP1 gene of *L. donovani* (B) Sequence alignment for locus C26882→T in the ankyrin gene of *L donovani*. (C) Sequence alignment for locus T527→A in the miltefosine transporter of *L donovani*. (D) Sequence alignment for locus C1259→A in the miltefosine transporter of *L donovani*. Accession number corresponds to the published sequences of the LdMT and LdRos3 genes obtained for the *L. donovani* strain MHOM/ET/1967/HU3 (Victoria et al., 2006). The reference strain (wild-type) (MHOM/IN/1983/AG83) is depicted in blue colour, and the reference strain (LdM30) (MHOM/IN/2009/BHU573M30) in which miltefosine resistance was experimentally induced in red colour. Further reference strain LdBPK282A1 represented in maroon. Identified point mutations are highlighted in yellow.
